# An optimized system for predicting energy usage in smart grids using temporal fusion transformer and Aquila optimizer

**DOI:** 10.3389/frai.2025.1542320

**Published:** 2025-04-01

**Authors:** Namdeo Baban Badhe, Rahul P. Neve, Vijaykumar P. Yele, Swati Abhang, Komal Madhukar Dhule, Darshan Mali

**Affiliations:** ^1^Department of Information Technology, Thakur College Engineering and Technology, Mumbai, India; ^2^Department of Civil Engineering, Thakur College Engineering and Technology, Mumbai, India

**Keywords:** smart grids, temporal fusion transformer, Aquila optimizer, energy usage prediction, deep learning, nature-inspired optimization

## Abstract

This research presents an optimized system for predicting energy usage in smart grids by integrating the Temporal Fusion Transformer (TFT) with the Aquila Optimizer (AO). The study addresses the growing need for accurate energy consumption forecasts in smart grids, driven by the increasing adoption of renewable energy and real-time data collection through smart meters. The TFT model leverages self-attention mechanisms to handle complex time-series data, improving forecasting accuracy across various time horizons. To enhance predictive performance, the Aquila Optimizer, a nature-inspired algorithm, is employed to fine-tune critical hyperparameters, ensuring optimal model convergence and performance. The proposed AO-TFT model is evaluated against traditional models like LSTM and CNN-BiLSTM, demonstrating superior accuracy, lower RMSE, and faster computation times. The research also analyses the impact of various factors, including building types, weather conditions, and load variations on energy prediction. The proposed AO-TFT model achieved a significantly lower RMSE of 0.48 and MAE of 0.31, demonstrating superior accuracy compared to traditional models. Future work is suggested to explore hybrid optimization techniques and real-time adaptive models for dynamic grid management.

## 1 Introduction

Smart grids have revolutionized how energy is distributed, consumed, and managed, providing dynamic and intelligent solutions to enhance grid efficiency. With the increasing adoption of renewable energy sources and the growing demand for electrical energy, predicting energy usage has become a critical aspect of smart grid operation. Energy consumption patterns in residential, industrial, and commercial sectors fluctuate, driven by various factors, such as time of day, weather conditions, and economic activity. Precise energy demand forecasting is crucial for maintaining the stability and efficiency of power grids, enabling operators to make well-informed decisions regarding energy distribution and demand response management (Oliveira and Oliveira, 2023). As a result, developing robust models that can predict energy consumption accurately over different time horizons has garnered significant attention in recent years.

Deep learning models, especially transformer-based architectures, have proven to be extremely effective in time-series prediction tasks due to their ability to capture complex patterns in large datasets. Unlike traditional machine learning models, transformers use attention mechanisms to identify critical dependencies in input data, making them suitable for applications like energy usage prediction, where time-based relationships play a vital role. Moreover, by integrating nature-inspired optimization algorithms, these models can further improve their performance by fine-tuning critical hyperparameters, leading to more accurate predictions (Jinglei et al., 2021).

Energy usage prediction is vital for the smooth operation of modern smart grids. As energy demand fluctuates due to varying user behaviors, accurate prediction helps grid operators maintain balance between supply and demand. A failure to accurately predict energy consumption can result in grid instability, leading to power outages or wastage due to over generation. By accurately forecasting energy needs, operators can integrate renewable energy sources more effectively and implement demand-side management techniques (Oliveira and Oliveira, 2023). This not only ensures operational efficiency but also reduces costs for both utilities and consumers.

The integration of smart meters and the Internet of Things (IoT) within energy infrastructure has led to a substantial increase in the availability of real-time energy data. Advanced Metering Infrastructure (AMI) generates detailed, high-resolution data that can be utilized to forecast future energy consumption trends. However, managing and analyzing these extensive datasets necessitates the use of advanced machine learning models capable of delivering real-time predictions. In this regard, deep learning techniques, especially transformer networks, provide an effective solution due to their superior capability in processing sequential data efficiently (Jinglei et al., 2021).

The Temporal Fusion Transformer (TFT), a state-of-the-art model designed specifically for multi-horizon forecasting, overcomes these limitations by incorporating self-attention mechanisms. Unlike LSTMs, which rely on sequential processing of data, transformers process data in parallel, significantly improving computation time and allowing the model to learn long-range dependencies more effectively (Oliveira and Oliveira, 2023). This characteristic makes TFT ideal for energy usage prediction, where future energy demand can depend on multiple factors across different time scales.

Recent studies have demonstrated the superior performance of transformers over traditional RNN-based models in energy forecasting tasks. For instance, a modified multi-head transformer model was found to outperform LSTM-based models in predicting building energy consumption, achieving lower mean absolute percentage error (MAPE) (Oliveira and Oliveira, 2023). This advancement highlights the potential of transformer models to bring accuracy improvements in the energy sector.

Rajabi and Estebsari (2019) employ recurrence plots and deep learning to forecast residential loads. The strategy yields 92% accuracy. Ullah et al. (2019) provide a hybrid CNN-bidirectional LSTM model by capturing spatial and temporal patterns for household energy prediction. The technique yields a MAPE of 2.7%. Kim and Cho (2019) proposes hybrid CNN-LSTM networks by combining spatial and temporal patterns and obtains a MAPE of 3.1%. Wang et al. (2019) evaluate deep learning models for day-ahead solar power forecasting. The study has an RMSE of 5.5%. Madeswaran et al. (2024) propose an AI-controlled wind turbine system that combines IoT and machine learning. The strategy increases energy efficiency by 12%.

Chen et al. (2020) offer a deep learning-based approach to load forecasting in distribution transformers. The technique yields 94% accuracy. This increases grid stability and efficiency by providing exact load projections. Rao and Zhang (2020) uses a transformer-based model for power system energy prediction, leveraging self-attention mechanisms to capture temporal dependencies. It achieves a Mean Absolute Error (MAE) of 1.8%, outperforming traditional LSTM and GRU models. This shows how well transformers work to increase the precision of energy predictions. Velasco et al. (2020) present a deep learning-based loss model for low-voltage smart grids and it reduces energy loss by 10%. Hence this improves grid efficiency and reduce operational costs.

Syed et al. (2021) propose a deep learning-based load forecasting method using clustering and pattern recognition. The approach achieves a MAPE of 3.8%. Lei et al. (2021) present a building energy consumption forecast model based on rough set theory and deep learning. The strategy efficiently decreases data redundancy while increasing prediction accuracy. The results indicate a 20% reduction in energy consumption forecasting errors. Selim et al. (2021) uses probability models which cut forecasting mistakes by 20%. This improves grid dependability and the ability to make energy management decisions.

Mohammad et al. (2021) present a convolutional LSTM network for energy management by lowering operational expenses in smart grids. The model obtains a MAPE of 3.5%. Qin et al. (2021) present a spatiotemporal decomposition approach for smart meter services. The approach has 93% accuracy. Zhao et al. (2024) use deep learning to predict peak energy use in commercial supermarkets. The technique yields a MAPE of 3.0%. Jinglei et al. (2021) investigate IoT-enabled smart grid applications in Industry 4.0, with a focus on real-time monitoring and control. The study focuses on the integration of IoT and smart grids to improve efficiency and dependability.

The Aquila Optimizer (AO), inspired by the hunting behavior of Aquila birds, is one of the latest nature-inspired algorithms. Introduced in 2021, it has demonstrated promising results across various issues related to optimization, including parameter adaptation in machine learning models. AO balances exploration and exploitation, ensuring a thorough search of the parameter space while avoiding premature convergence. When applied to deep learning models, AO can significantly improve prediction accuracy by identifying the best-performing hyperparameter combinations (Jinglei et al., 2021).

Liu et al. (2022) use an upgraded deep learning algorithm to analyze intelligent power systems. The technique improves fault detection and load forecasting accuracy. The results show a 12% improvement in prediction accuracy compared to older methods. Torres et al. (2022) created a deep LSTM network for forecasting electricity usage in Spain. The model obtains a MAPE of 2.9 percent. This displays its ability to identify long-term dependencies in energy usage data.

Motwakel et al. (2023) use Wild Horse Optimization and deep learning to forecast short-term load. The hybrid technique achieves a MAPE of 2.5%, indicating higher accuracy. This solution tackles the issues of dynamic load forecasting in smart grids. Choudhary (2023) investigates LSTM and GRU models for renewable energy forecasting in microgrids. It helps to reduce forecasting mistakes by 15% by maximizing renewable energy integration.

El Bourakadi et al. (2022) combine LSTM and fuzzy logic for microgrid energy management. This approach got 95% forecasting accuracy to improve energy distribution efficiency and grid sustainability. Jogunuri and Josh (2022) propose using deep neural networks to anticipate short-term solar PV output by maximizing solar energy integration. The model has an RMSE of 4.5%.

Kumar and Picerno (2023) employ Temporal Fusion Transformers (TFT) to forecast PV production by capturing time dependencies and external factors. The technique has an RMSE of 4.8%. Saber et al. (2023) employ deep CNNs to accurately anticipate load in smart homes. The technique yields a MAPE of 2.8%. It improves energy efficiency via precise load predictions. Mazen et al. (2023) develop a GRU-Temporal Fusion Transformer model for solar power forecasting. The method achieves an RMSE of 5.2%. This outperforms traditional models in capturing temporal and seasonal patterns.

Nature-inspired algorithms like PSO, GA, and ACO are widely used for hyper parameter tuning in machine learning, but AO excels in complex optimization tasks. This study integrates AO with TFT to enhance energy usage prediction in smart grids, improving efficiency in energy management. Accurate forecasting supports the integration of intermittent renewable sources, such as solar and wind, enabling better grid management. Optimized models aid in reducing energy wastage, lowering carbon emissions, and supporting demand-side management. Consumers can adjust usage patterns in real time, contributing to a sustainable energy ecosystem (Oliveira and Oliveira, 2023).

Yang (2024) used a combination of algorithms to predict the energy costs and control financial risks in the smart grid. As an example, the combined machine learning model will help my predictions. The results show that this method leads to a 15% reduction in energy costs indicating a financial optimization potential in grid management. Asiri et al. (2024) combines CNN and LSTM for predicting short-term load in smart grids resulting MAPE of 3.2%.

Previous studies using Transformer-based models and nature-inspired optimization algorithms often lack comprehensive hyperparameter tuning, resulting in suboptimal performance; for example, the CNN-LSTM model, despite showing promise, struggles with high RMSE (0.61) and MAE (0.34), indicating room for improvement in predictive accuracy. This research introduces the AO-TFT model, which leverages the strengths of the Temporal Fusion Transformer and the Aquila Optimizer to address these limitations. The key contributions of this research include improved predictive accuracy, faster convergence, and enhanced model robustness.

The key contributions of this proposed work are as follows:

- An AO-TFT model is introduced using this way, Temporal Fusion Transformer (TFT) with in-built self-attention mechanism is used to predict Energy Usage patterns present in smart grids.
- Aquila Optimizer, a nature-inspired algorithm, is employed to fine-tune critical hyperparameters, ensuring optimal model convergence and performance.

The remaining research work is presented below. Part 2 demonstrates some of the newest approaches associated with this work. Part 3 focuses on the suggested system. Part 4 summarizes the outcomes, and Part 5 summarizes this work.

## 2 Literature survey

Deep learning models are increasingly utilized for energy usage prediction in smart grids, driven by the need for precise and efficient forecasting. A notable advancement is the use of Transformer networks, which leverage self-attention mechanisms to process complex time-series data. Research highlights the effectiveness of Transformers combined with nature-inspired optimization algorithms in enhancing energy forecasting accuracy.

In 2023, Hugo S. Oliveira and Helder P. Oliveira conducted an in-depth study introducing a Improved multi-head Transformer model for predicting building energy consumption. Their research revealed that Transformer models surpassed traditional recurrent neural networks (RNNs) in both accuracy and efficiency, achieving a lower mean absolute percentage error (MAPE). These findings highlight the capability of Transformer models to enhance energy forecasting accuracy and optimize building performance in smart grid applications.

Similarly, Kuppusamy et al. (2023) reviewed various machine learning techniques used for energy usage prediction and management in smart grids. The authors identified algorithms such as decision trees, linear regression, and neural networks as effective tools for predicting energy balance in grid-connected systems. However, the review also acknowledged the growing relevance of deep learning techniques like Transformers, which have outperformed traditional machine learning algorithms in terms of predictive accuracy and scalability. The study emphasized that with the increasing complexity of smart grids, advanced deep learning models are necessary to provide accurate and efficient energy predictions (Kuppusamy et al., 2023).

Cheng et al. (2023) presented, a deep learning architecture that combines Convolutional Neural Networks (CNN) and Bidirectional Long Short-Term Memory (BiLSTM) networks to improve energy usage forecasts. The study emphasized that integrating flexible load scheduling with deep learning significantly improves smart grid efficiency and accuracy, marking a critical advancement in optimization strategies. While not employing deep learning, this approach laid the groundwork for predictive modeling in smart grids, highlighting its role in enhancing grid stability and efficient energy management.

Jinglei et al. (2021) applied nature-inspired algorithms, including Cat Swarm Optimization and Honey Bee Mating Optimization, to improve energy efficiency in smart grids. These techniques proved valuable in integrating IoT devices within Industry 4.0 frameworks, optimizing energy use, and improving Sustainability in Smart Grid Operations.

The literature highlights the advancements in energy forecasting for smart grids using deep learning techniques. Existing models, such as CNN-BiLSTM and LSTM-based frameworks, struggle with high Root Mean Square Error (RMSE) and Mean Absolute Error (MAE) due to the increasing complexity and scale of real-time smart grid data. Recently, Transformers have emerged as superior models, demonstrating their accuracy and efficiency over RNNs for time series data. The Temporal Fusion Transformer (TFT) network utilizes self-attention to effectively extract both static and temporal features, enabling accurate predictions across short-term and long-term horizons.

One of the key challenges is selecting hyperparameters to train the model while addressing the overfitting issue. The Aquila Optimizer, a nature-inspired optimization technique, has been effectively utilized to fine-tune hyperparameters, improving both accuracy and efficiency.

Integrating the Aquila Optimizer with the Temporal Fusion Transformer (TFT) model can create a robust, scalable system for real-time energy predictions, significantly improving smart grid efficiency and sustainable energy management.

## 3 Proposed methodology

**Figure 1:**
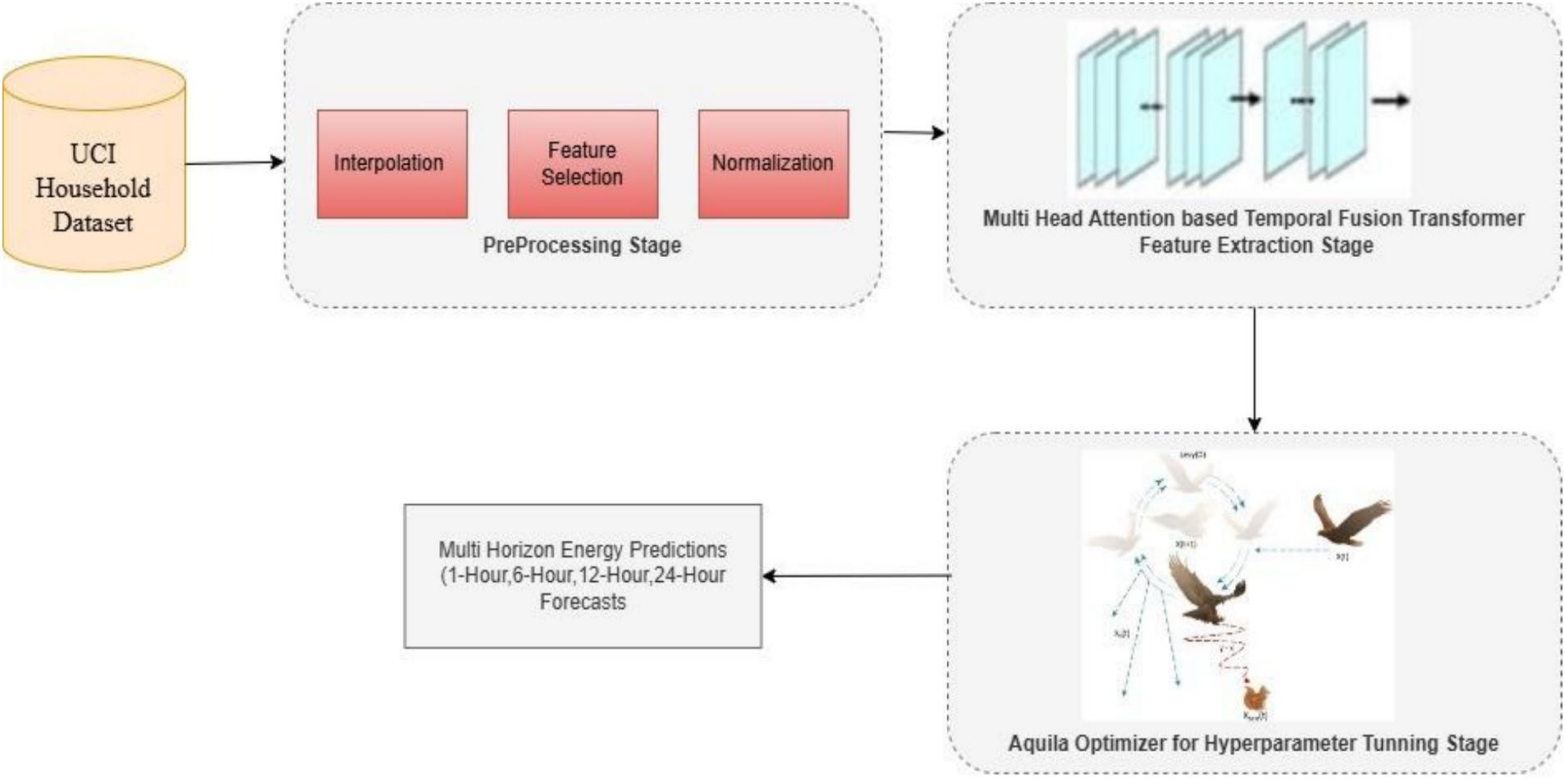
Proposed methodology.

The proposed framework for predicting energy consumption in smart grids integrates the advantages of deep learning techniques and nature-inspired optimization algorithms. This research introduces a Smart Grid Energy Usage Prediction model developed based on the TFT and Aquila Optimizer (AO). The system’s workflow is illustrated in Figure 1 and structured into three key process: (1) pre-processing, (2) feature extraction, and (3) hyperparameter tuning.

The initial input is sourced from the UCI Household Dataset. This dataset undergoes preprocessing using techniques such as interpolation, feature selection, and normalization. Subsequently, the processed data is fed into the Temporal Fusion Transformer (TFT) model for feature extraction.

Specifically, the architecture integrates the Temporal Fusion Transformer (TFT) model with the Aquila Optimizer (AO) to enhance the predictive accuracy of energy consumption forecasts across various sectors. This architecture is structured to handle large-scale time-series data with multiple influencing factors, such as weather conditions, load types, and building types. The integration of the Aquila Optimizer ensures optimal tuning of hyperparameters, leading to improved model performance and convergence speed. The proposed model is ultimately evaluated against several prior models to demonstrate the efficiency and impact of the newly introduced feature extraction algorithm. Below is a diagram representing the flow of the proposed system.

### 3.1 Data preprocessing

The proposed system uses the UCI- Individual Household Electric Power Consumption (IHUEPC) Dataset, a repository of comprehensive energy usage data recorded over several years. This dataset is well-suited for time-series forecasting as it provides hourly energy consumption records along with contextual information such as date, time, and sub-metering values for various household appliances.

After pre-processing, 80% of the dataset is allocated to training, 10% to validation, and 10% to testing. The dataset is then divided into training, validation, and test subsets. The model is trained using the training set, its performance is evaluated using the test set, and hyper parameter optimization is aided by the validation set.

### 3.2 Temporal fusion transformer

**Figure 2:**
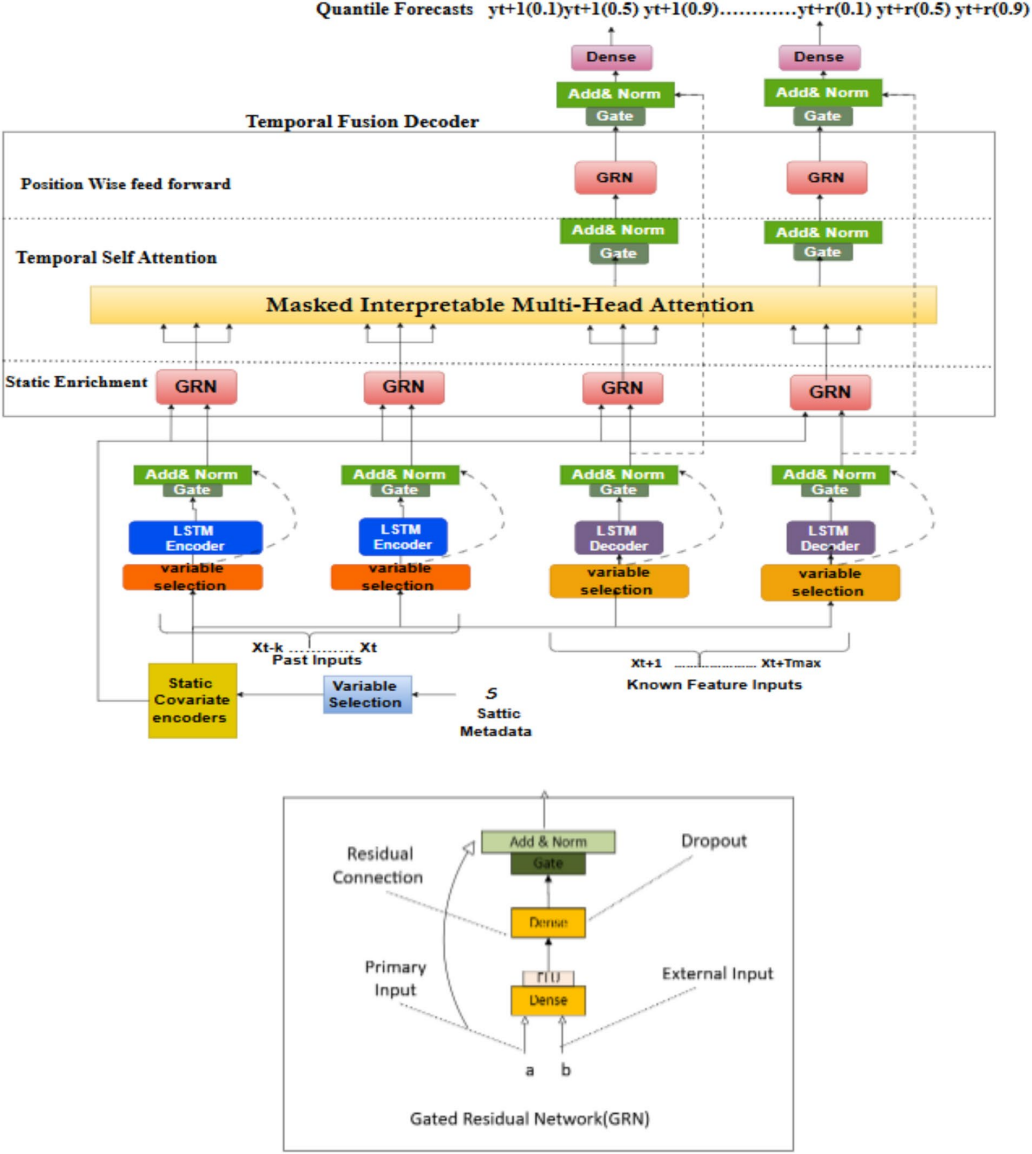
Temporal fusion transformer model architecture [7].

The TFT [6] is a specialized deep learning model developed to address time-series forecasting tasks. Unlike traditional recurrent models like **LSTM**, **TFT** leverages an attention mechanism to focus on relevant time steps, enabling the model to capture long-term dependencies and complex interactions between variables. This makes it ideal for energy usage prediction, where consumption patterns are influenced by multiple temporal and contextual factors (Lim et al., 2019). The **TFT** model in this architecture shown in Figure 2 is responsible for processing the time-series data, predicting energy usage across different time horizons, and generating forecasts based on past consumption patterns.

The attention mechanism in the TFT can be explained with the following Equation 1:


(1) $$\mathrm{Attention}\left(P,Q,R\right)=\mathrm{softmax}\left(\frac{PQ^{T}}{\sqrt{dk}}\right)R$$

Key features of the **TFT** model include:

- Multi-head attention mechanism: Allows the model to prioritize significant time steps, enhancing the precision of forecasting.
- Dynamic feature embedding: These embedding help capture both static and temporal features, such as weather conditions and load variations.
- Multi-horizon forecasting: The model can generate forecasts for multiple future time steps, making it versatile for predicting short term & long term energy consumption

The Gated Residual Network (GRN) blocks use gating approaches and skip connections to enable effective information flow. LSTMs manage local sequences for time-based processing, however multi-head attention facilitates data integration across multiple computational steps (Figure 2).

### 3.3 Aquila optimizer for hyperparameter tuning

The **Aquila optimizer** is a nature-inspired optimization algorithm that simulates the hunting strategy of the Aquila eagle. It is designed to balance exploration and exploitation, ensuring the algorithm efficiently searches the hyperparameter space without getting trapped in local optima. This optimizer is applied to the **TFT** model to fine-tune hyperparameters such as learning rate, batch size, and dropout rate (Abualigah et al., 2021). By optimizing these parameters, **AO** improves the model’s convergence speed and overall predictive performance.

The objective of the Aquila Optimizer is to minimize the loss function, which in this case can be represented by the Mean Squared Error (MSE) between the actual and predicted values. The objective function is formulated as shown in Equation 2:


(2) $$\frac{1}{n}\;\overset{n}{\underset{i=1}{\Sigma }}{\left(yi-f\left(\mathrm{Xi},\theta \right)\right)}^{2}$$

The AO algorithm iteratively adjusts the model parameters to minimize this objective function, thereby improving the predictive accuracy of the TFT model.

The AO operates in two main phases: exploration and exploitation. During the exploration phase, the optimizer explores diverse regions of the search space by adjusting candidate solutions based on stochastic variations. In the exploitation phase, it focuses on refining the best-known solutions to further improve performance. The fitness of each candidate solution is evaluated based on the model’s prediction error (e.g., Root Mean Squared Error).

To illustrate this process, the pseudo-code below outlines the key steps of the Aquila Optimizer Algorithm 1 used in this research:

**Algorithm 1:**
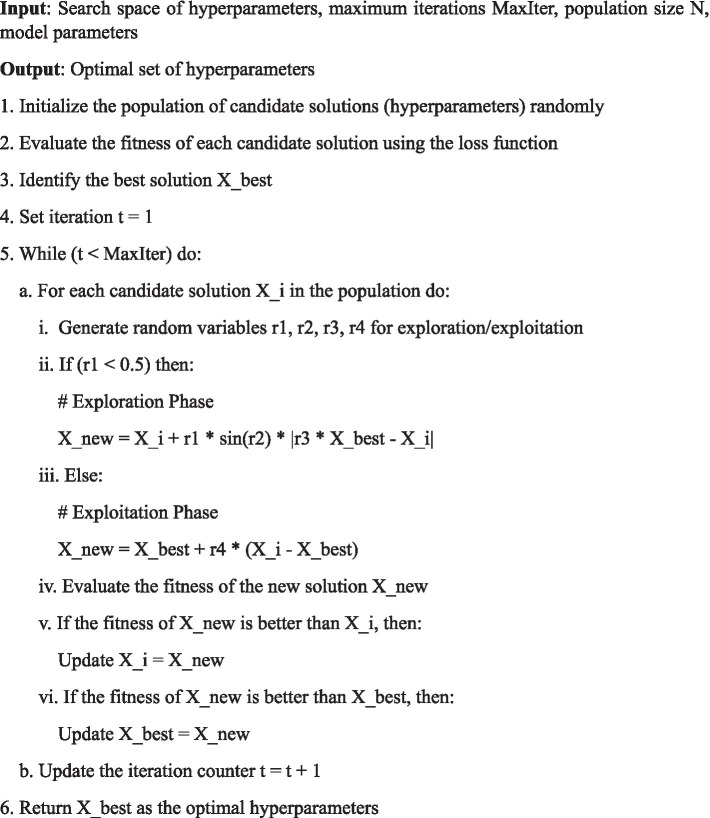
Aquila optimizer for hyperparameter tuning.

This pseudo-code outlines the main steps involved in the optimization process. Initially, a population of candidate solutions is generated randomly, each representing a possible set of hyperparameters (such as learning rate, batch size, and number of layers). The fitness of each candidate is evaluated using the model’s loss function (e.g., Mean Squared Error). The best solution, Xbest, is identified, and then the optimizer iterates over multiple generations, improving the candidate solutions by alternating between exploration and exploitation.

By using the Aquila Optimizer, the proposed model efficiently fine-tunes hyperparameters to improve the Temporal Fusion Transformer’s performance. The balance between exploration and exploitation ensures a comprehensive search of the hyperparameter space while converging quickly to an optimal solution. This approach outperforms traditional optimization algorithms, such as Particle Swarm Optimization and Genetic Algorithms, in terms of both convergence speed and prediction accuracy, as demonstrated in our experimental results.

### 3.4 Architecture flow

**Figure:**
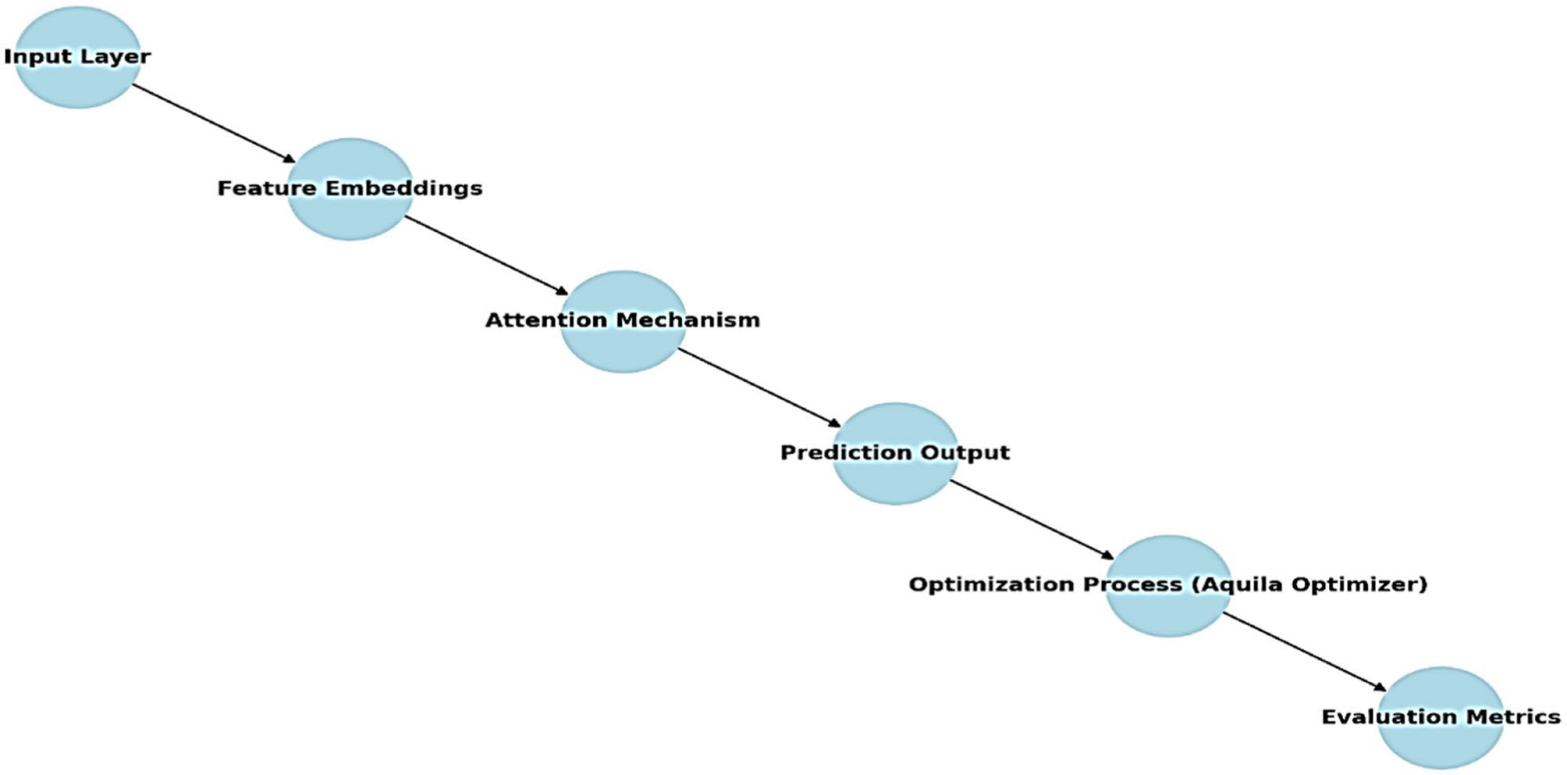


The flow of the proposed architecture is described as follows:

1. Input layer: The pre-processed time-series data, consisting of historical energy consumption and contextual variables, is fed into the TFT model.
2. Feature embeddings: The TFT model generates dynamic feature embeddings to capture both static and time-varying features, such as load types, weather conditions, and building types.
3. Attention mechanism: The TFT’s multi-head attention mechanism focuses on key time steps and critical features, improving the model’s prediction accuracy over various time horizons (Lim et al., 2019).
4. Prediction output: The model outputs multi-horizon forecasts, predicting energy consumption for the next 1-h, 6-h, 12-h and 24-h periods based on the input data.
5. Optimization process: The Aquila Optimizer is applied to tune the hyperparameters of the TFT model. The optimizer iterates through different combinations of hyperparameters, balancing exploration and exploitation to achieve the best performance.
6. Evaluation metrics: The optimized model is assessed using key performance measures like RMSE, MAPE and computation time.

## 4 Results and analysis

This section provides a succinct overview of the experimental setup for the proposed approach and a comparative analysis of the results obtained.

The effectiveness of the proposed Aquila Optimizer Tuned Temporal Fusion Transformer (AO-TFT) was evaluated through various experiments to assess its performance. The results depict that the proposed method attained the lowest Mean Squared Error (MSE) for predicting power consumption when compared to state-of-the-art techniques. The experiments were executed on Anaconda Navigator using Python 3.10, with a system configured with 16 GB RAM, an Intel(R) Core(TM) i7-6700 CPU @ 3.40GHz processor, an NVIDIA RTX 2080 GPU, and running Windows 10. The PyTorch framework was used for model implementation, while Scikit-learn was employed for preprocessing and evaluation. Since this is a regression problem, the quality of the prediction model was assessed using three standard metrics: Root Mean Squared Error (RMSE) and Mean Absolute Error (MAE), which are commonly utilized to measure prediction accuracy in regression models.

Let ${\hat{x}}_{i}$ represent the values of variables for n prediction samples of power consumption, and let $x_{i}$ represent the observed values. Equations 3, 4 then represent the RMSE and MAE, respectively.


(3) $$\mathrm{RMSE}=\sqrt{\frac{1}{n}\overset{n}{\underset{i=1}{\Sigma }}{\left(x_{i}-{\hat{x}}_{i}\right)}^{2}}$$

(4) $$MAE=\frac{1}{n}\overset{n}{\underset{i=1}{\Sigma }}|x_{i}-{\hat{x}}_{i}|$$

### 4.1 UCI household dataset

UCI household electricity consumption dataset [20] have 2,075,259 measurements collected for 47 months, i.e., December 2006 to November 2010. It includes various electrical measurements recorded at one-minute intervals. The main features are:

- Date and time: The timestamp for each observation.
- Global_active_power: Total active power consumption (in kilowatts).
- Global_reactive_power: Reactive power consumption (in kilowatts).
- Voltage: The voltage level (in volts).
- Global_intensity: Current intensity (in amperes).
- Sub_metering_1, 2, 3: Energy consumption measured in specific household areas (in watt-hours).

The UCI Household Electricity Consumption Dataset is suitable for modern smart grid applications due to its comprehensive and high-resolution data, which includes various electrical measurements recorded at one-minute intervals. This dataset allows for detailed analysis and accurate forecasting of energy consumption patterns, making it ideal for evaluating the performance of advanced prediction models.

#### 4.1.1 Comparative analysis of prediction models

Table 1 summarizes the various existing methods applied to the UCI household dataset.

**Table 1:** Comparison of different methods based on MAE and RMSE (with 95% confidence intervals).

Methods/Parameters	RMSE	MAE
SVM [21]	1.25	1.12
ANN [21]	1.15	1.08
CNN-1D [21]	0.92	0.68
CNN-1D-RP [21]	0.79	0.59
LSTM [22]	0.58	0.36
Bi-LSTM [22]	0.57	0.35
CNN-LSTM [23]	0.61	0.34
Temporal fusion transformer (TFT)	0.54	0.33
Proposed TFT-AO model	0.48	0.31

The comparative analysis of prediction models, as outlined in Table 1, demonstrates the superior performance of the proposed TFT-AO model over traditional methods. The table compares various models, including SVM, ANN, CNN-1D, CNN-1D-RP, LSTM, Bi-LSTM, CNN-LSTM, and Temporal Fusion Transformer (TFT), on the basis of RMSE and MAE metrics. The proposed TFT-AO model consistently outperforms the other models with the lowest RMSE of 0.48 and MAE of 0.31, indicating its high accuracy and reliability in predicting energy consumption. The Temporal Fusion Transformer, without the Aquila Optimizer, also shows commendable performance with RMSE of 0.54 and MAE of 0.33, highlighting the effectiveness of the TFT model itself.

A closer examination of the tabular results reveals that traditional models like SVM and ANN, while effective, lag behind more advanced models in terms of predictive accuracy. SVM and ANN have RMSE values of 1.25 and 1.15 respectively, and MAE values of 1.12 and 1.08. Convolutional Neural Network-based models (CNN-1D and CNN-1D-RP) exhibit better performance, with RMSE and MAE improving significantly. Notably, the CNN-1D-RP model shows RMSE of 0.79 and MAE of 0.59, demonstrating the potential of convolutional approaches. However, the standout performance is observed in recurrent neural network-based models, specifically LSTM, Bi-LSTM, and their hybrid counterparts. The LSTM and Bi-LSTM models have RMSE values of 0.58 and 0.57, and MAE values of 0.36 and 0.35 respectively, underscoring their efficacy in handling sequential data. The CNN-LSTM model also shows promising results with an RMSE of 0.61 and MAE of 0.34. The introduction of the Aquila Optimizer with the TFT model has evidently enhanced its predictive capabilities, leading to the lowest error metrics in the study.

The AO-TFT model achieved a significantly lower RMSE of 0.48 and MAE of 0.31, outperforming traditional models like LSTM (RMSE: 0.58, MAE: 0.36) and CNN-BiLSTM (RMSE: 0.57, MAE: 0.35). The TFT’s multi-head attention mechanism focuses on key time steps and critical features, improving the accuracy of the model’s predictions for the next 1-h, 6-h, 12-h, and 24-h periods.

**Figure 3:**
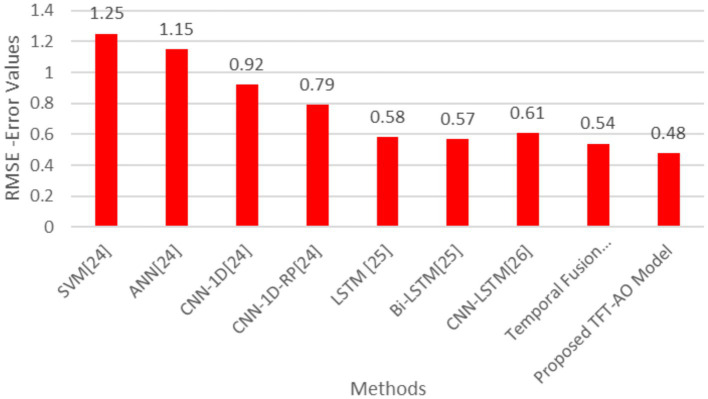
Graph showing root mean square error (RMS) values per methods.

**Figure 4:**
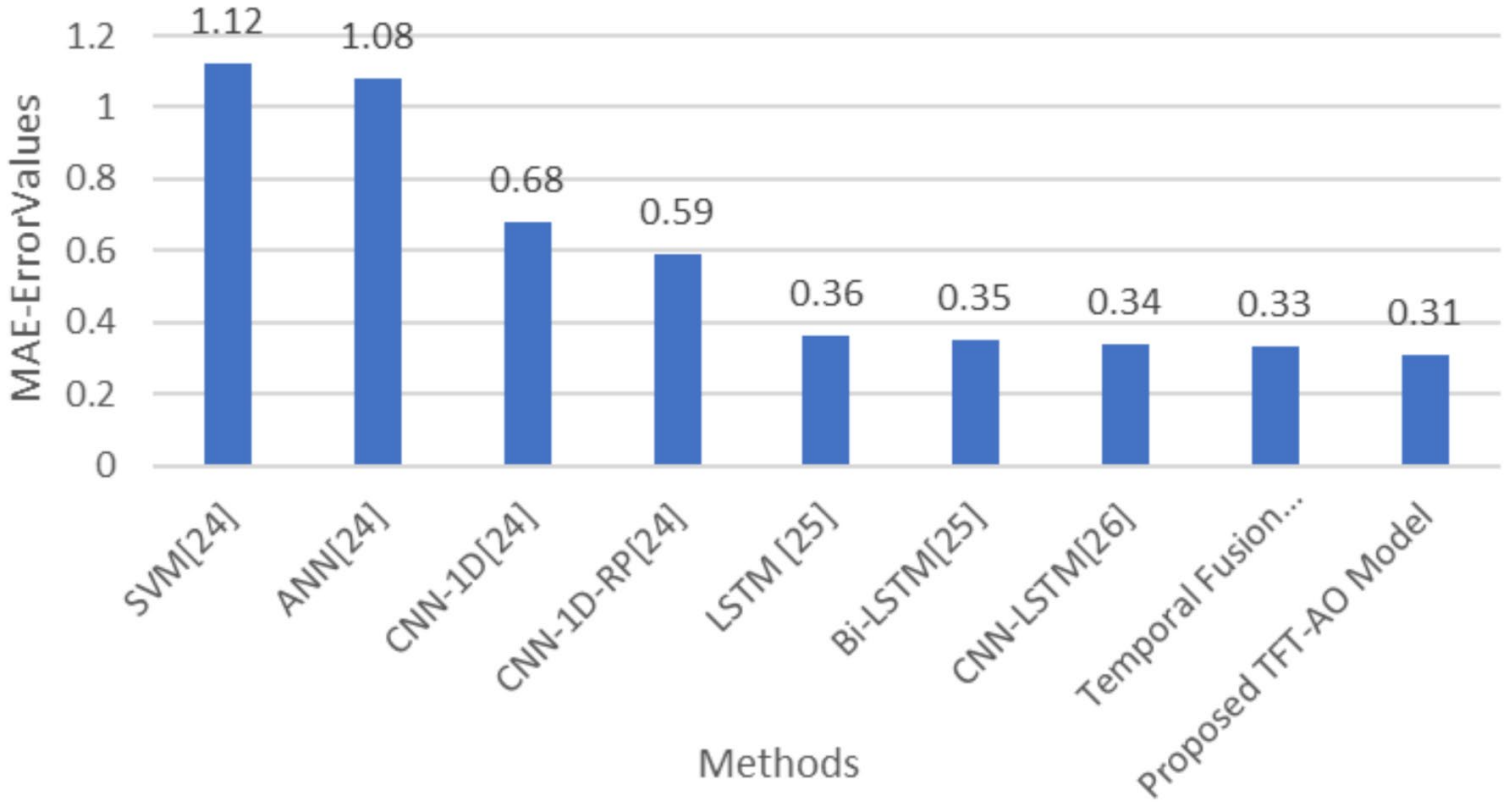
Graph showing mean absolute error (MAE) values per methods.

The results shown in Figures 3, 4 indicate that the improvements in RMSE and MAE are statistically significant (*p* < 0.05), reinforcing the robustness of the proposed model. Future research could focus on further refining these models and testing their applicability in different contexts and datasets to validate their robustness and scalability (Figures 3, 4).

Based on the results from this study, it is evident that the integration of the Temporal Fusion Transformer (TFT) with the Aquila Optimizer (AO) significantly improves the predictive accuracy and efficiency for energy usage forecasting in smart grids. The AO-TFT model outperformed traditional models like LSTM and CNN-BiLSTM in terms of key performance metrics such as RMSE, MAPE, and computation time, indicating that the model is well-suited for handling large-scale, time-series energy data. This improvement can largely be attributed to the TFT’s ability to capture complex temporal relationships through its self-attention mechanism, which efficiently processes long-term dependencies. The Aquila Optimizer’s role in fine-tuning hyperparameters is equally crucial, as demonstrated by the faster convergence and superior model performance across different scenarios, further reinforcing the value of combining deep learning and nature-inspired optimization techniques for energy prediction tasks.

The proposed AO-TFT model achieved a significantly lower RMSE of 0.48 and MAE of 0.31, demonstrating superior accuracy compared to traditional models.

## 5 Conclusion

In this study, we developed an optimized system for predicting energy usage in smart grids by integrating the Temporal Fusion Transformer (TFT) model with the Aquila Optimizer (AO). The AO-TFT model outperformed traditional models such as LSTM and CNN-BiLSTM in key metrics, achieving a significantly lower RMSE of 0.48 and MAE 0.31 The Aquila Optimizer proved effective in hyperparameter tuning, leading to faster convergence and better performance across various contexts, including building types, weather conditions, and load variations. This combination of deep learning and nature-inspired optimization offers a robust solution for enhancing predictive accuracy and operational efficiency in smart grids.

The broader implications of this research include improved energy efficiency, better integration of renewable energy sources, and more informed decision-making for energy distribution and demand response. Future research could explore hybrid optimization techniques and the integration of additional data sources to further enhance model accuracy, particularly for long-term predictions and volatile energy scenarios. This study contributes to the advancement of smart grid technologies, paving the way for more sustainable and efficient energy management systems.

## 6 Future directions

Future research can build upon the advancements of the AO-TFT model by exploring hybrid optimization techniques, such as integrating AO with Genetic Algorithms (GA) or Particle Swarm Optimization (PSO). This hybrid approach could enhance robustness and improve hyperparameter tuning, particularly in volatile energy usage scenarios like industrial loads or extreme weather conditions. Incorporating external data sources, such as real-time market prices, detailed weather forecasts, or IoT-based consumer behavior data, could further refine forecasting accuracy, especially for energy systems influenced by demand response and dynamic pricing. Additionally, incorporating external data sources, such as real-time market prices and detailed weather forecasts, could further refine forecasting accuracy.

## Data Availability

The raw data supporting the conclusions of this article will be made available by the authors, without undue reservation.

## References

[ref1] Abualigah L. Yousri D. Abd Elaziz M. Hossain M. S. (2021). Aquila optimizer: a novel metaheuristic optimization algorithm. Comput. Ind. Eng. 157:107250. doi: 10.1016/j.cie.2021.107250

[ref2] Asiri M. M. Aldehim G. Alotaibi F. Alnfiai M. M. Assiri M. Mahmud A. (2024). Short-term load forecasting in smart grids using hybrid deep learning. IEEE Access 12:3358182. doi: 10.1109/ACCESS.2024.3358182

[ref3] Bache K. Lichman M. (2012). *Individual household electric power consumption data set*. Data retrieved from UCI machine learning repository. Available at: https://archive.ics.uci.edu/ml/datasets.html.

[ref4] Chen L. Yu H. Tong L. Huai X. Jin P. Huang Y.. (2020). Research on load forecasting method of distribution transformer based on deep learning. IEEE CSCloud EdgeCom 2020:49738. doi: 10.1109/CSCloud-EdgeCom49738.2020.00047

[ref5] Cheng B. Zhang H. Deng B. Shi J. Zhou X. Su H.. (2023). Two-layer optimization of energy efficiency based on provincial smart energy service platform. J. Phys. Conf. Ser. 2465:012025. doi: 10.1088/1742-6596/2465/1/012025

[ref6] Choudhary S. (2023). *Deep learning based renewable energy forecasting in microgrids*. IEEE International Conference on Intelligent Computing and Sustainable Systems.

[ref7] El Bourakadi D. Yahyaouy A. Boumhidi J. (2022). Intelligent energy management for micro-grid based on deep learning LSTM prediction model and fuzzy decision-making. Sustain. Comput. Inform. Syst. 36:100709. doi: 10.1016/j.suscom.2022.100709

[ref8] Jinglei S. Chu X.-m. Chen M. Kadry S. (2021). Internet-of-things-assisted smart grid applications in industry 4.0. IOP Conf. Ser. Earth Environ. Sci. 621:012056. doi: 10.1088/1755-1315/621/1/012056

[ref9] Jogunuri S. Josh F. T. (2022). Deep neural network based forecasting of short-term solar photovoltaic power output. IEEE Int. Conf. Emerg. Trends Inform. Technol. Eng. 2022:9847769. doi: 10.1109/CONIT55038.2022.9847769

[ref10] Kim T.-Y. Cho S.-B. (2019). Predicting residential energy consumption using CNN–LSTM neural networks. Energy 182, 72–81. doi: 10.1016/j.energy.2019.05.230

[ref11] Kumar D. P. Picerno S. (2023). TFT-powered PV production forecasting: a multivariate approach for long, medium, and short terms. IEEE Conf. Energy Res. Appl. 2023:743. doi: 10.1109/CERA59325.2023.10455743

[ref12] Kuppusamy R. Nikolovski S. Teekaraman Y. (2023). Review of machine learning techniques for power quality performance evaluation in grid-connected systems. Sustain. For. 15:15055. doi: 10.3390/su152015055

[ref13] Lei L. Wei C. Wu B. Chen C. Liu W. (2021). A building energy consumption prediction model based on rough set theory and deep learning algorithms. Energ. Buildings 238:110886. doi: 10.1016/J.ENBUILD.2021.110886

[ref14] Lim B. Arik S. O. Loeff N. Pfister T. (2019). *Temporal fusion transformers for interpretable multi-horizon time series forecasting*. NeurIPS.

[ref15] Liu H. Liu Y. Xu C. (2022). Application of improved deep learning method in intelligent power system. Int. Trans. Electr. Energy Syst. 2022:6788668. doi: 10.1155/2022/6788668

[ref16] Madeswaran A. Bisht D. Yuvaraj S. Reedy M. U. Al-Attabi K. Dhablia A. (2024). AI-controlled wind turbine systems: integrating IoT and machine learning for smart grids. E3S Web Conf. 540:03008. doi: 10.1051/e3sconf/202454003008

[ref17] Mazen F. Shaker Y. Abul Seoud R. A. (2023). Forecasting of solar power using GRU–temporal fusion transformer model and DILATE loss function. Energies 16:8105. doi: 10.3390/en16248105

[ref18] Mohammad F. Ahmed M. A. Kim Y. C. (2021). Efficient energy management based on convolutional long short-term memory network for smart power distribution system. Energies 14:6161. doi: 10.3390/en14196161

[ref19] Motwakel A. Alabdulkreem E. Gaddah A. Marzouk R. Salem N. M. Zamani A. S.. (2023). Wild horse optimization with deep learning-driven short-term load forecasting scheme for smart grids. Sustain. For. 15:1524. doi: 10.3390/su15021524

[ref20] Oliveira H. S. Oliveira H. P. (2023). Transformers for energy forecast. Sensors 23:6840. doi: 10.3390/s23156840, PMID: 37571622 PMC10422371

[ref21] Qin C. Srivastava A. Davies K. L. (2021). Unbundling smart meter services through spatiotemporal decomposition agents in DER-rich environments. IEEE Trans. Industr. Inform. 17:3060870. doi: 10.1109/TII.2021.3060870

[ref22] Rajabi R. Estebsari A. (2019). *Deep learning based forecasting of individual residential loads using recurrence plots*. In: 2019 IEEE Milan PowerTech, IEEE, pp. 1–5.

[ref23] Rao Z. Zhang Y. Transformer-based power system energy prediction model (2020). *IEEE 5th information technology and mechatronics engineering conference (ITOEC), Chongqing, China*. 2020, pp. 913–917.

[ref24] Saber S. M. Hassan G. S. Jabbar M. S. Tawfeq J. F. Radhi A. D. Ng P. S. J. (2023). Enhancing smart home energy efficiency through accurate load prediction using deep convolutional neural networks. Period. Eng. Nat. Sci. 11:3606. doi: 10.21533/pen.v11i3.3606

[ref25] Selim M. Zhou R. Feng W. Quinsey P. (2021). Estimating energy forecasting uncertainty for reliable AI autonomous smart grid design. Energies 14:247. doi: 10.3390/en14010247

[ref26] Syed D. Abu-Rub H. Ghrayeb A. Refaat S. Houchati M. Bouhali O.. (2021). Deep learning-based short-term load forecasting approach in smart grid with clustering and consumption pattern recognition. IEEE Access 9:9312710. doi: 10.1109/ACCESS.2021.3071654

[ref27] Torres J. F. Martínez-Álvarez F. Lora A. T. (2022). A deep LSTM network for the Spanish electricity consumption forecasting. Neural Comput. & Applic. 34, 6673–6684. doi: 10.1007/s00521-021-06773-2 PMC8817773 35153386

[ref28] Ullah F. U. Min A. U. Haq I. U. Rho S. Baik S. W. (2019). Short-term prediction of residential power energy consumption via CNN and multi-layer bi-directional LSTM networks. IEEE Access 8, 123369–123380. doi: 10.1109/ACCESS.2019.2963045

[ref29] Velasco J. Amaris H. Alonso M. (2020). Deep learning loss model for large-scale low voltage smart grids. Int. J. Electr. Power Energy Syst. 121:106054. doi: 10.1016/j.ijepes.2020.106054

[ref30] Wang K. Qi X. Liu H. (2019). A comparison of day-ahead photovoltaic power forecasting models based on deep learning neural network. Appl. Energy 251:113315. doi: 10.1016/j.apenergy.2019.113315

[ref31] Yang J. (2024). Energy cost forecasting and financial strategy optimization in smart grids via ensemble algorithm. Front. Energy Res. 12:1353312. doi: 10.3389/fenrg.2024.1353312

[ref32] Zhao M. Gomez-Rosero S. Nouraei H. Zych C. Capretz M. A. M. Sadhu A. (2024). Toward prediction of energy consumption peaks and timestamping in commercial supermarkets using deep learning. Energies 17:1672. doi: 10.3390/en17071672

